# Breast Cancer: Mitochondria-Centered Metabolic Alterations in Tumor and Associated Adipose Tissue

**DOI:** 10.3390/cells13020155

**Published:** 2024-01-15

**Authors:** Tamara Zakic, Andjelika Kalezic, Zorka Drvendzija, Mirjana Udicki, Tatjana Ivkovic Kapicl, Biljana Srdic Galic, Aleksandra Korac, Aleksandra Jankovic, Bato Korac

**Affiliations:** 1 Institute for Biological Research “Sinisa Stankovic”—National Institute of Republic of Serbia, University of Belgrade, 11000 Belgrade, Serbia; tamara.zakic@ibiss.bg.ac.rs (T.Z.); andjelika.kalezic@ibiss.bg.ac.rs (A.K.); aleksandra.jankovic@ibiss.bg.ac.rs (A.J.); 2 Faculty of Medicine, University of Novi Sad, 21000 Novi Sad, Serbia; zorka.drvendzija@mf.uns.ac.rs (Z.D.); mirjana.udicki@mf.uns.ac.rs (M.U.); biljana.srdic-galic@mf.uns.ac.rs (B.S.G.); 3 Oncology Institute of Vojvodina, 21204 Sremska Kamenica, Serbia; tatjana.ivkovic-kapicl@mf.uns.ac.rs; 4 Faculty of Biology, University of Belgrade, 11000 Belgrade, Serbia; aleksandra.korac@bio.bg.ac.rs

**Keywords:** breast cancer, mitochondria, metabolism

## Abstract

The close cooperation between breast cancer and cancer-associated adipose tissue (CAAT) shapes the malignant phenotype, but the role of mitochondrial metabolic reprogramming and obesity in breast cancer remains undecided, especially in premenopausal women. Here, we examined mitochondrial metabolic dynamics in paired biopsies of malignant versus benign breast tumor tissue and CAAT in normal-weight and overweight/obese premenopausal women. Lower protein level of pyruvate dehydrogenase and citrate synthase in malignant tumor tissue indicated decreased carbon flux from glucose into the Krebs cycle, whereas the trend was just the opposite in malignant CAAT. Simultaneously, stimulated lipolysis in CAAT of obese women was followed by upregulated β-oxidation, as well as fatty acid synthesis enzymes in both tumor tissue and CAAT of women with malignant tumors, corroborating their physical association. Further, protein level of electron transport chain complexes was generally increased in tumor tissue and CAAT from women with malignant tumors, respective to obesity. Preserved mitochondrial structure in malignant tumor tissue was also observed. However, mitochondrial DNA copy number and protein levels of PGC-1α were dependent on both malignancy and obesity in tumor tissue and CAAT. In conclusion, metabolic cooperation between breast cancer and CAAT in premenopausal women involves obesity-related, synchronized changes in mitochondrial metabolism.

## 1. Introduction

Breast cancer is the most common cancer in women worldwide, with very heterogenous molecular, histopathological, and clinical patterns [1]. In recent years, the tumor microenvironment (TME), and in particular the interplay between cancer cells and TME components, has been recognized as one of the major factors in tumorigenesis. The most prominent component of the breast cancer microenvironment is cancer-associated adipose tissue (CAAT) with a highly altered phenotype [2,3,4]. Cancer cells affect CAAT via paracrine signaling, and vice versa: affected CAAT highly influences tumor initiation, progression, and metastasis through secreted adipocytokines, as well as metabolic crosstalk [4].

Breast cancer is nowadays considered a metabolic disease where intratumor heterogeneity and immense metabolic plasticity underly heterogenous metabolic profiles of cancer cells. The relationship between breast cancer and CAAT results in mutual, cancer-driven metabolic reprogramming necessary to meet increasing bioenergetic and biosynthetic demands [5,6,7]. Our previous studies focused on understanding the relationship between cancer tissue and CAAT in premenopausal women with breast cancer and the influence of obesity on this crosstalk. We demonstrated tissue-specific parallel reprogramming of glucose and lactate metabolism of cancer tissue and CAAT coordinated with their distinct redox profiles, revealing malignancy and/or obesity-related redox–metabolic cooperation between breast cancer and adipose tissue [8,9], which was also in line with other studies [10,11].

While it is well established that cancer cells mostly rely on glycolysis (Warburg effect), recent data point out that they can have different metabolic preferences depending on the microenvironment conditions, such as nutrient and oxygen availability [6,12]. To fully utilize the metabolites derived from the adipocyte-rich microenvironment, breast cancer cells most likely switch from typically glucose-centered to lipid-centered metabolism thus mostly relying on mitochondrial β-oxidation/oxidative phosphorylation for ATP production. The lipid-centered connection between breast cancer and adipose tissue is particularly pronounced in obesity [8,9,13,14]. Thus, understanding the molecular mechanisms underlying metabolic communication between cancer cells and adipocytes would help understand the impact obesity has on breast cancer progression.

Substantial studies on solid tumors, including breast cancer, corroborated that cancer cells alter rather than inactivate mitochondrial oxidative metabolism, which is favored during tumor migration/invasion and metastasis [6]. In this regard, mitochondrial metabolic reprogramming is imperative for the metabolic plasticity of cancer cells. Further, breast cancer cells deprived of mitochondrial DNA (mtDNA) displayed diminished or no tumorigenesis, which was only restored after the reintroduction of mtDNA [15,16,17]. However, the exact role of mitochondrial metabolism in breast cancer progression is still controversial.

Overall, breast cancer is characterized by extensive reprogramming of metabolic pathways in both cancer tissue and CAAT. So far, the alterations in mitochondrial metabolism have been altogether overlooked as a crucial element of breast cancer tumorigenesis. The impact of obesity on cancer progression, especially in premenopausal women, is still under debate considering limited data on humans. Therefore, this study aimed to further dissect the breast cancer–CAAT connection in premenopausal women, focusing on mutual metabolic reprogramming primarily associated with mitochondria and relative to malignancy and/or obesity. To this end, we investigated enzymes involved in oxidative decarboxylation of pyruvate, Krebs cycle, fatty acid β-oxidation (and synthesis), and oxidative phosphorylation, as well as the parameters of mitochondrial dynamics—mitochondrial DNA copy number (MCN), peroxisome proliferator-activated receptor gamma coactivator-1 alpha (PGC-1α), and mitochondrial structure—in breast tumor tissue and mammary adipose tissue from premenopausal women with benign or malignant tumors.

## 2. Materials and Methods

### 2.1. Patient Recruitment and Sample Collection

All procedures were approved by The Ethics Committee of the Clinical Center of Vojvodina (reference number: 4/19/1-1486/2-13). Participants involved in this study were thirty-six premenopausal women with diagnosed benign or malignant breast tumors who had had regular menstrual cycles for the last six months. All of the patients signed an informed consent form and were previously scheduled for surgical removal of breast tumors. During surgical interventions, breast tumor tissue and tumor-associated adipose tissue categorized as breast adipose tissue in the proximity of the tumor but outside of its invasive front were obtained. Histopathological analysis classified benign breast tumors as fibroadenomas and malignant breast tumors as invasive ductal carcinomas (luminal type A, ER^+^/PR^+^/HER2^−^). Following the body mass index (BMI), patients were grouped into normal-weight (BMI < 25 kg/m^2^) or overweight/obese (BMI ≥ 25 kg/m^2^), and thus, also in regard to breast tumor classification, forming 4 experimental groups (*n* = 9): normal-weight with benign tumors, overweight/obese with benign tumors, normal-weight with malignant tumors, and overweight/obese with malignant tumors. Patients did not have chronic diseases, were not smokers, did not consume alcohol, and had no family history of breast cancer. For details on patients’ characteristics, please see our previous paper Kalezic et al. [8]. Each obtained tissue sample was immediately snap-frozen in liquid nitrogen and stored at −80 °C until subsequent DNA and protein isolation using TRI Reagent (Thermo Fisher Scientific, Waltham, MA, USA) for Western blot and RT-PCR analyses.

### 2.2. Western Blotting

Proteins from the tissue samples were obtained using Trizol reagent according to the manufacturer’s protocol (Invitrogen, Life Technologies, Waltham, MA, USA). Western blot analysis for protein levels was performed according to the previously described procedure [18]. Primary antibodies against target proteins were purchased from Abcam (Cambridge, UK) and Santa Cruz (Dallas, TX, USA), and used at the following concentrations: β-actin (ab8226, 0.5 μg/mL), PGC-1α (ab54481, 1 μg/mL), citrate synthase (CS; sc-390693, 1 μg/mL), Complex I (ab55521, 0.5 μg/mL), Complex II (ab14715, 0.5 μg/mL), Complex III (ab14745, 0.2 μg/mL), Complex IV (ab14744, 0.5 μg/mL), ATP synthase (ab14730, 1 μg/mL), pyruvate dehydrogenase (PDH; ab84588, 1 μg/mL), pyruvate dehydrogenase kinase 4 (PDK4; ab89295, 1 μg/mL), acyl-CoA dehydrogenase medium chain (ACADM; ab92461, 1 μg/mL), acyl-CoA oxidase 1 (ACOX1; ab184032, 1 μg/mL), acetyl-CoA carboxylase (ACC; ab45174, 0.5 μg/mL), fatty acid synthase (FAS; ab150508, 0.5 μg/mL), adipose triglyceride lipase (ATGL; sc-365278, 1 μg/mL). Just before gel loading, the samples from each group (*n* = 9) were pooled/combined by three into three final samples per group due to the small quantity of the samples. The whole blots for each target protein are shown in the figures (for the original blots, also see Supplementary Figure S1). Immunoreactive bands were quantified using ImageJ software (Version v1.53c, National Institutes of Health, Bethesda, MD, USA). The final band densities were determined as the sum of pixel intensities per band averaged against gel loading control (β-actin). The protein content is displayed as average protein level in arbitrary units (AU) from three independent experiments.

### 2.3. Mitochondrial DNA Copy Number

After total DNA extraction with Trizol (Invitrogen, Waltham, MA, USA), and the assessment of DNA concentration and purity with NanoPhotometer^®^ (Implen GmbH, Munich, Germany), 20 ng of total DNA was used for the RT-PCR replication of the nuclear 18S gene and mitochondrial ND2 gene. The reaction mixture contained 5 pM corresponding primers, fluorescent dye SYBR Green with reference fluorescent dye ROX (Applied Biosystems, Waltham, MA, USA), TaqMan polymerase, and 20 ng of total DNA. The reaction was performed under the following cycling conditions using QuantStudioTM 3 ReaL-Time PCR System (Thermo Fischer Scientific, Waltham, MA, USA): initial denaturation at 95 °C for 5 min, 40 cycles of denaturation at 95 °C for 15 s, primer annealing at 60 °C for 60 s, and elongation at 72 °C for 60 s. Relative mitochondrial copy number in breast tumor and adipose tissue samples was calculated as mitochondrial DNA content relative to nuclear DNA content. Commercially available gene sequences for 18S and ND2, as well as the sequences of the primers used for their replication, were as follows: 18S: 5′-TAGAGGGACAAGTGGCGTTCAGCCACCCGAGATTGAGCAATAACAGGTCTGTGATGCCCTTAGATGTCC-3′; ND2: 5′-ACTGCGCTAAGCTCGCACTGATTTTTTACCTGAGTAGGCCTAGAAATA AACATGCTAGCTTTTATTCCA-3′; 18S: (F) 5′-TAGAGGGACAAGTGGCGT-3′, (R) 5′-CGCTGAGCCAGTCAGTGT-3′; ND2: (F) 5′-ACTGCGCTAAGCTCGCACTGA-3′, (R) 5′-GATTATGGATGCGGTTGCTTG-3′. 

### 2.4. Light and Electron Microscopy

Breast cancer tissue samples were prepared for electron and light microscopy analysis. Briefly, routinely formalin-fixed and paraffin-embedded tissue samples were used for re-embedding in resin for electron microscopy (AGR1141, Agar Sci, Essex, UK). Further, resin blocks of tissue samples were cut in 1 μm or 80 nm thick sections using a Leica UC6 ultramicrotome (Leica Microsystems, Wetzlar, Germany), mounted on glass slides or copper grids, and stained with basic fuchsine and methylene blue or contrasted using UA-Zero (Agar Sci, Essex, UK), respectively. Sections were examined on an optical light microscope (Leica DLMB, Leica Microsystems, Wetzlar, Germany) or a Philips CM12 transmission electron microscope (Philips/FEI, Eindhoven, The Netherlands) equipped with a digital camera (SIS MegaView III, Olympus Soft Imaging Solutions, Münster, Germany).

### 2.5. Statistical Analyses

GraphPad Prism software (Version 8.4.3 GraphPad Software, San Diego, CA, USA) was utilized to conduct all statistical analyses. All datasets were assessed for the normality of distribution via D’Agostino or Pearson’s omnibus normality tests. The evaluation of intergroup variations was determined with a two-way analysis of variance (ANOVA) followed by multiple comparisons Tukey’s post-hoc test. Error bars represent the standard error of the mean (S.E.M). Statistical significance was accepted at *p* < 0.05.

## 3. Results

### 3.1. Contrasting Profiles of Major Metabolic Pathways in Breast Cancer Tissue and CAAT

**Figure 1:**
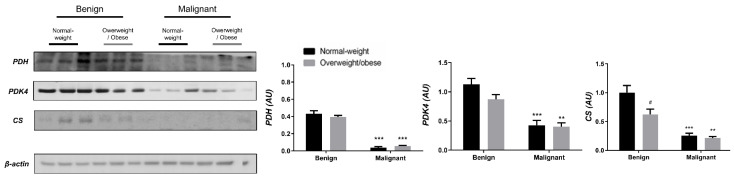
The protein levels of PDH, PDK4, and CS in benign and malignant **breast tumor tissue** from normal-weight (black) and overweight/obese (gray) women. The protein level is normalized to β-actin protein levels and expressed in arbitrary units (AU). The graphs display mean values of densitometric analysis for each group (*n* = 3). Three bands representing each group are displayed for every target protein; each band denotes pooled samples from three different patients. Bars represent mean values ± S.E.M. Asterisks Comparison to respective benign counterparts, ** *p* < 0.01, *** *p* < 0.001. Hashtag Comparison to respective normal-weight counterparts, # *p* < 0.05.

**Figure 2:**
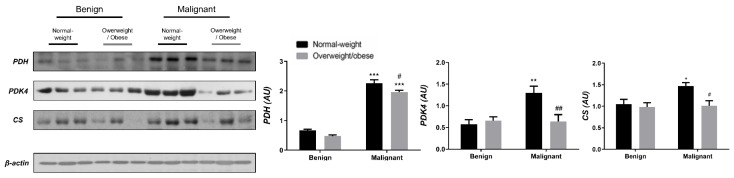
The protein levels of PDH, PDK4, and CS in **CAAT** from normal-weight (black) and overweight/obese (gray) women with benign and malignant breast tumor tissue. The protein level is normalized to β-actin protein levels and expressed in arbitrary units (AU). The graphs display mean values of densitometric analysis for each group (*n* = 3). Three bands representing each group are displayed for every target protein; each band denotes pooled samples from three different patients. Bars represent mean values ± S.E.M. Asterisks Comparison to respective benign counterparts, * *p* < 0.05, ** *p* < 0.01, *** *p* < 0.001. Hashtag Comparison to respective normal-weight counterparts, # *p* < 0.05, ## *p* < 0.01.

Examining the protein level of key enzymes involved in the oxidative decarboxylation of pyruvate and the Krebs cycle revealed major differences between benign and malignant tumor tissue, independently from obesity. Both PDH and PDK4 protein levels were significantly lower and obesity-independent in malignant tumor tissue compared to benign tumor tissue. The protein level of the first enzyme in the Krebs cycle, CS, was also decreased and obesity-independent in malignant tumor tissue compared to benign tumor tissue, all supporting decreased glucose flux through the Krebs cycle in malignancy (Figure 1). In contrast, the protein level of PDH in CAAT of women with malignant tumors was higher compared to CAAT of women with benign tumors, irrespective of obesity. Moreover, PDK4 protein level was higher only in CAAT of normal-weight women with malignant tumors compared to CAAT of normal-weight women with benign tumors. Similarly, the protein level of CS was increased only in normal-weight women with malignant tumors compared to normal-weight women with benign tumors. Interestingly, CAAT of obese women with malignant tumors showed lower levels of examined proteins compared to their normal-weight malignant counterparts, pointing to the impact of obesity on CAAT metabolism in malignancy (Figure 2).

### 3.2. Mitochondrial Metabolism in Breast Cancer Is Maintained on the Grounds of Lipid Metabolism

**Figure 3:**
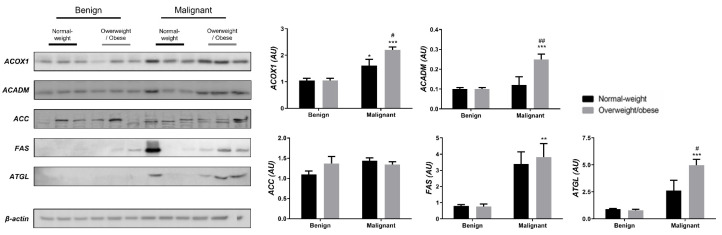
The protein levels of ACOX1, ACADM, ACC, FAS, and ATGL in benign and malignant **breast tumor tissue** from normal-weight (black) and overweight/obese (gray) women. The protein level is normalized to β-actin protein levels and expressed in arbitrary units (AU). The graphs display mean values of densitometric analysis for each group (*n* = 3). Three bands representing each group are displayed for every target protein; each band denotes pooled samples from three different patients. Bars represent mean values ± S.E.M. Asterisks Comparison to respective benign counterparts, * *p* < 0.05, ** *p* < 0.01, *** *p* < 0.001. Hashtag Comparison to respective normal-weight counterparts, # *p* < 0.05, ## *p* < 0.01.

**Figure 4:**
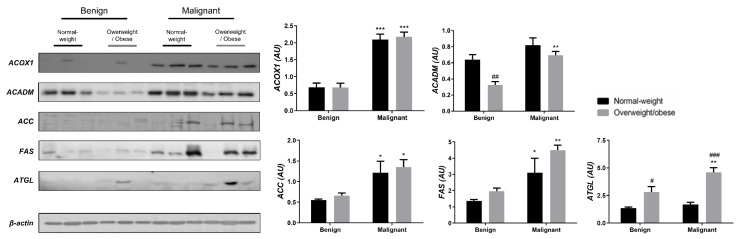
The protein levels of ACOX1, ACADM, ACC, FAS, and ATGL in **CAAT** from normal-weight (black) and overweight/obese (gray) women with benign and malignant breast tumor tissue. The protein level is normalized to β-actin protein levels and expressed in arbitrary units (AU). The graphs display mean values of densitometric analysis for each group (*n* = 3). Three bands representing each group are displayed for every target protein; each band denotes pooled samples from three different patients. Bars represent mean values ± S.E.M. Asterisks Comparison to respective benign counterparts, * *p* < 0.05, ** *p* < 0.01, *** *p* < 0.001. Hashtag Comparison to respective normal-weight counterparts, # *p* < 0.05, ## *p* < 0.01, ### *p* < 0.001.

We examined the protein level of the main enzymes involved in lipolysis, fatty acid synthesis, and β-oxidation in tumor tissue (Figure 3) and associated adipose tissue (Figure 4) of normal-weight and obese women to establish their lipid-revolved metabolic features. A similar pattern was seen in the protein level of lipolytic enzyme ATGL and β-oxidation enzymes, ACOX1 and ACADM, whose protein levels were higher in malignant tumor tissue of obese women compared to malignant tumor tissue of normal-weight women but also compared to their benign counterparts. The protein level of ACOX1 was additionally higher in malignant tumor tissue of normal-weight women compared to normal-weight women with benign tumors. There were no differences in the protein level of ACC relevant to malignancy or obesity. However, the FAS protein level was higher in malignant tumor tissue of obese women compared to benign tumor tissue of obese women, with the same trend in normal-weight women, supporting upregulated de novo fatty acid synthesis during tumor growth. In CAAT, ATGL protein level was increased in obese women with benign and malignant tumors compared to their normal-weight counterparts. Similarly, ATGL protein level was even more increased in obese women with malignant tumors compared to obese women with benign tumors. Increased lipolysis in adipose tissue supported upregulated β-oxidation enzymes in tumor tissue, especially in obesity. β-oxidation was stimulated in CAAT as well, since the protein levels of ACOX1 were higher in CAAT of women with malignant tumors compared to women with benign tumors irrespective of obesity, while ACADM protein level was higher only in obese women with malignant tumors compared to obese women with benign tumors. Fatty acid synthesis was significantly upregulated in CAAT of women with malignant tumors, independent from obesity since it was characterized by increased protein level of both ACC and FAS.

### 3.3. Obesity-Affected Oxidative Phosphorylation in Mitochondria Is a Major Constituent of Breast Cancer Progression

**Figure 5:**
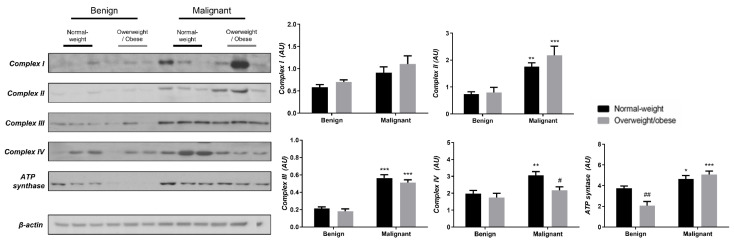
The protein levels of Complex I, Complex II, Complex III, Complex IV, and ATP synthase in benign and malignant **breast tumor tissue** from normal-weight (black) and overweight/obese (gray) women. The protein level is normalized to β-actin protein levels and expressed in arbitrary units (AU). The graphs display mean values of densitometric analysis for each group (*n* = 3). Three bands representing each group are displayed for every target protein; each band denotes pooled samples from three different patients. The bars represent mean values ± S.E.M. Asterisks Comparison to respective benign counterparts, * *p* < 0.05, ** *p* < 0.01, *** *p* < 0.001. Hashtag Comparison to respective normal-weight counterparts, # *p* < 0.05, ## *p* < 0.01.

**Figure 6:**
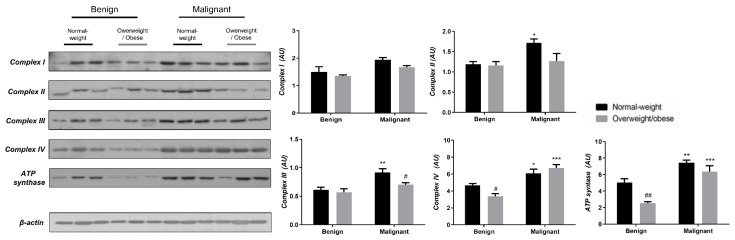
The protein levels of Complex I, Complex II, Complex III Complex IV, and ATP synthase in **CAAT** from normal-weight (black) and overweight/obese (gray) women with benign and malignant breast tumor tissue. The protein level is normalized to β-actin protein levels and expressed in arbitrary units (AU). The graphs display mean values of densitometric analysis for each group (*n* = 3). Three bands representing each group are displayed for every target protein; each band denotes pooled samples from three different patients. Bars represent mean values ± S.E.M. Asterisks Comparison to respective benign counterpart, * *p* < 0.05, ** *p* < 0.01, *** *p* < 0.001. Hashtag Comparison to respective normal-weight counterpart, # *p* < 0.05, ## *p* < 0.01.

To reveal the significance of oxidative metabolism for tumorigenesis, we performed a Western blot analysis of electron transport chain (ETC) complexes and ATP synthase in breast cancer tissue and CAAT of normal-weight and obese women. The differences in the protein level of ETC complexes and ATP synthase between benign and malignant tumor tissue of normal-weight and obese women are depicted in Figure 5. No significant differences were found in the protein level of Complex I relevant to malignancy or obesity. However, the protein levels of Complex II, Complex III, and ATP, synthase were higher in malignant tumor tissue compared to benign tumor tissue, independently from obesity. The protein level of Complex IV is dependent on obesity; not only it is higher in malignant tumor tissue compared to benign tumor tissue, but it is also lower in malignant tumor tissue of obese women compared to malignant tumor tissue of normal-weight women. In the same manner, we found differences in the protein level of ETC complexes and ATP synthase between CAAT of normal-weight and obese women with benign/malignant tumors (Figure 6). No significant differences were found in the protein level of Complex I relevant to malignancy or obesity. However, the protein levels of Complex II and III were higher in CAAT of normal-weight women with malignant tumors compared to the respective CAAT of women with benign tumors, highlighting the impact of malignancy on the presence of mitochondrial metabolic reprogramming of mammary adipose tissue. The protein levels of Complex IV and ATP synthase were higher in CAAT of women with malignant tumors compared to CAAT of women with benign tumors, independently from obesity, with their lower protein level in CAAT of obese women with benign tumors compared to CAAT of normal-weight women with benign tumors, highlighting the impact of obesity on the reprogramming of mammary adipose tissue premenopausal in women with benign tumors.

### 3.4. Mitochondrial Dynamics in Breast Tumor Tissue and CAAT

**Figure 7:**
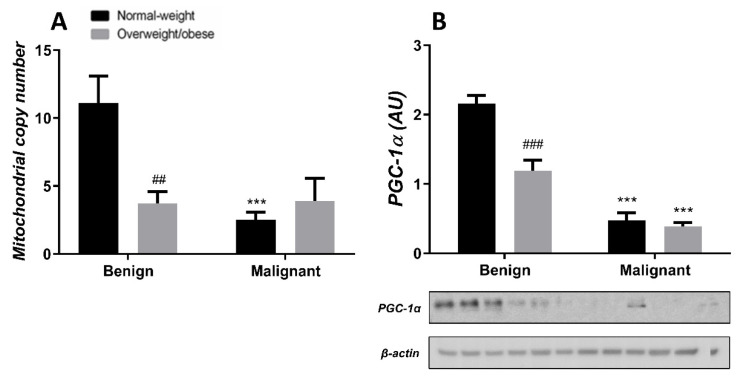
MCN (**A**) and protein level of PGC-1α (**B**) in benign and malignant **breast tumor tissue** from normal-weight (black) and overweight/obese (gray) women. The level of mitochondrial genome expression is shown as a relation between the expression of mitochondrial transcript for leucine tRNA and nuclear transcript for 18S rRNA. The protein level is normalized to β-actin protein levels and expressed in arbitrary units (AU). The graphs display mean values of densitometric analysis for each group (*n* = 3). Three bands representing each group are displayed for every target protein; each band denotes pooled samples from three different patients. Bars represent mean values ± S.E.M. Asterisks Comparison to respective benign counterparts, *** *p* < 0.001. Hashtag Comparison to respective normal-weight counterparts, ## *p* < 0.01, ### *p* < 0.001.

**Figure 8:**
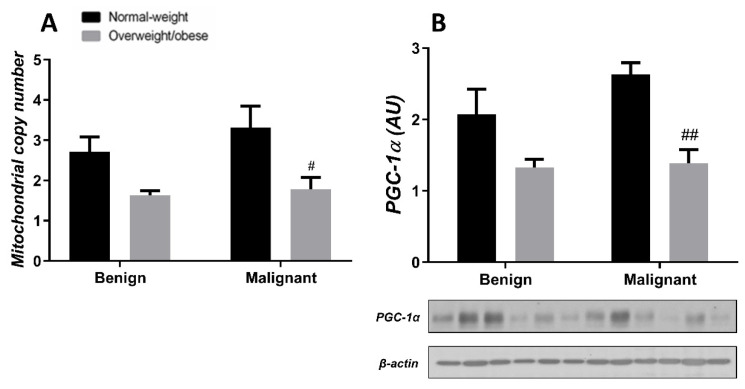
MCN (**A**) and protein level of PGC-1α (**B**) in **CAAT** from normal-weight (black) and overweight/obese (gray) women with benign and malignant breast tumor tissue. The level of mitochondrial genome expression is shown as a relation between the expression of mitochondrial transcript for leucine tRNA and nuclear transcript for 18S rRNA. The protein level is normalized to β-actin protein levels and expressed in arbitrary units (AU). The graphs display mean values of densitometric analysis for each group (*n* = 3). Three bands representing each group are displayed for every target protein; each band denotes pooled samples from three different patients. Bars represent mean values ± S.E.M. Hashtag Comparison to respective normal-weight counterparts, # *p* < 0.05, ## *p* < 0.01.

Generally, the level of MCN in malignant tumor tissue is maintained at a low level, irrespective of obesity. MCN is lower in normal-weight women with malignant tumors compared to normal-weight women with benign tumors. Also, MCN is lower in benign tumor tissue of obese women compared to normal-weight women. The protein level of PGC-1α is entirely in line with MCN in both normal-weight and obese women with benign or malignant tumors (Figure 7). In CAAT, there is a trend of lower MCN in obese women with both benign and malignant women compared to normal-weight women. In CAAT, the protein level of PGC-1α is also lower in obese women with malignant tumors compared to normal-weight women with malignant tumors, while it is not different compared to obese women with benign tumors. The same trend of PGC-1α protein level can be seen in women with benign tumors (Figure 8).

### 3.5. Microscopy Revealed Tight Connection between Breast Cancer Cells and Associated Adipocytes and Preservation of Mitochondrial Structure in Malignancy

**Figure 9:**
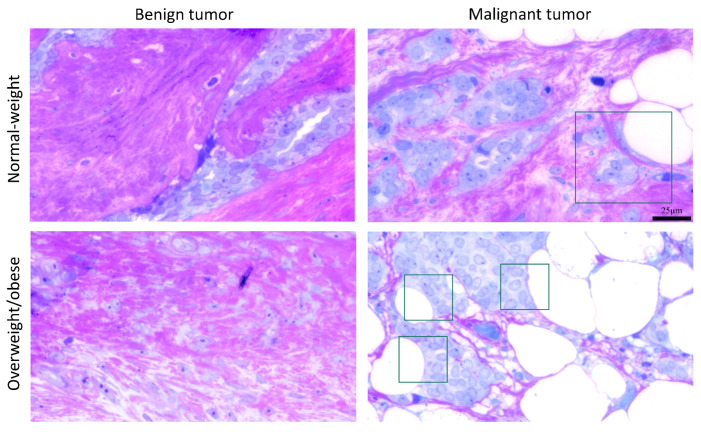
A representative light micrograph of tumor tissue showing the interaction of breast cancer cells and mammary adipocytes only in malignant tumors, regardless of obesity. The frame marks the interaction site. Magnification: ×100, orig. Bar: 25 µm.

**Figure 10:**
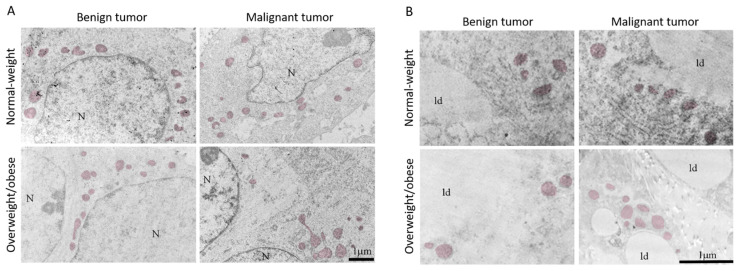
Representative electron micrographs of benign and malignant tumor tissue (**A**) and CAAT (**B**) from normal-weight and obese women showing the presence of preserved mitochondrial population (colored) in all groups. N: nucleus; ld: lipid droplet. Bar: 1 µm.

The light microscopy showed a tight physical connection between breast cancer cells and mammary adipocytes only in women with malignant tumors, in contrast to benign tumors where no similar morphological indications were seen (Figure 9). This connection between cancer cells and adipocytes is especially pronounced in obese women with malignant tumors. Further, both breast tumor tissue and associated mammary adipose tissue depict preserved mitochondrial populations (Figure 10), corroborating the maintenance of mitochondrial oxidative metabolism in breast cancer.

## 4. Discussion

Malignant tumor tissue and CAAT are characterized by tissue-specific strategies for metabolic reprogramming. In this study, we investigated the earmarks of mitochondrial metabolism reprogramming in breast cancer and CAAT from normal-weight and overweight/obese premenopausal women. While malignant tumor tissue showed lower protein levels of PDH, CS, and PDK4 in both normal-weight and obese women, CAAT showed an increase in the protein levels of the same enzymes with the exception of PDH in normal-weight women. Further, we found increased protein levels of ETC complexes (II, III, IV) and ATP synthase in malignant tumor tissue, independent of obesity, while in CAAT, this increase was obesity-related only in Complex II and III. Oxidative phosphorylation in tumor tissue was maintained on the grounds of upregulated lipid metabolism. Namely, higher ATGL protein level characterized the CAAT of obese women with malignant and benign tumors compared to their normal-weight counterparts, supporting the hypothesis of induced lipolysis in obesity. Stimulated lipolysis in malignant CAAT supported upregulated peroxisomal and mitochondrial β-oxidation in both CAAT and tumor tissue since they generally displayed increased protein level of ACOX1 and ACADM. These changes were followed by upregulated FAS protein levels in these tissues, pointing to increased fatty acid synthesis. In benign tumor tissue, we detected lower MCN in obese women compared to normal-weight women. A comparably lower level of MCN was found in malignant tumor tissue as well, independently from obesity. Similarly, we found a significantly lower MCN in CAAT of obese women with malignant tumors compared to the corresponding normal-weight group, with the same trend in women with benign tumors. The protein level of PGC-1α was consistent with MCN in tumor tissue, as well as in CAAT, demonstrating its pivotal role in mitochondrial dynamics. Overall, changes in the protein levels examined in this study suggest mitochondrial metabolic reprogramming of both breast cancer and associated adipose tissue affected by obesity, leading to tissue-specific phenotypes that characterize malignant transformation.

The malignant phenotype, including breast cancer initiation, implies not only the reprogramming of glycolysis but also other major metabolic pathways. Our previous results on premenopausal women with breast cancer showed that glucose metabolism in tumor tissue is directed toward glycolysis with lactate production and pentose phosphate pathway, while in CAAT it is primarily directed toward pentose phosphate pathway, displaying tissue-specific Warburg effect [9]. Additionally, synchronized changes in the expression of proteins involved in lactate metabolism pointed to the lactate-driven metabolic cooperation between breast cancer and associated adipose tissue [8]. Increased glycolysis is usually followed by decreased pyruvate derived from glucose in the Krebs cycle [19]. Thus, inhibition of PDH activity leads to the constitution of glycolytic phenotype, regulated particularly by the isoform PDK4 [20,21,22]. Accordingly, our results suggest lower glucose-derived pyruvate flux into the Krebs cycle since malignant tumor tissue had lower protein level of PDH compared to benign tumor tissue, independently from obesity. Additionally, malignant tumor tissue also displayed lower protein level of CS, the first enzyme in the Krebs cycle. In contrast, the CAAT of normal-weight women with malignant tumors showed increased protein level of PDH and CS, pointing to the metabolic cooperation between cancer cells and associated adipocytes [3]. Increased metabolic demands in cancer could be responsible for obesity-related mitochondrial dysfunction since there were no changes in the protein level of PDH and citrate synthase in the CAAT of obese women with malignant tumors. These results are in line with previously described lower protein level of CS in obese mice [23] and adipose tissue of obese patients [24,25]. Thus, metabolic reprogramming in breast cancer is affected by the influence of obesity on the tumor microenvironment.

Even though glycolysis is the predominant metabolic pathway for obtaining energy in malignant tumor tissue, changes in the mitochondrial metabolism of breast cancer possibly occur on the grounds of fatty acid metabolism reprogramming, including fatty acid storage/mobilization, β-oxidation, and de novo fatty acid synthesis. Cancer cells obtain fatty acids from either de novo lipid synthesis or TME to sustain tumor growth. It has been shown that breast cancer cells stimulate ATGL and HSL activity in adipocytes, promoting lipolysis and fatty acid release, which are then taken up by cancer cells, stored in complex lipids, or oxidized, thus causing increased proliferation, all of which is especially pronounced in obesity [13]. Similarly, the uptake of fatty acids from adipocytes supported the proliferation of cancer cells in colon cancer and acute monocyte leukemia [26,27]. Our results supported the proposed cooperation between cancer cells and adipocytes, where stimulated lipolysis in adipocytes supported upregulated β-oxidation in malignant tumor tissue, especially in obese women. CAAT of obese women with malignant tumors displayed higher ATGL protein level compared to obese women with benign tumors, corroborating the hypothesis of obesity-related induced lipolysis in malignancy.

Recent data on exosomes support lipid-centered communication between cancer cells and adipocytes, since adipocyte-derived exosomes mainly transfer lipids together with the proteins involved in fatty acid β-oxidation [28,29,30]. Following, we showed increased protein levels of β-oxidation enzymes in malignant CAAT: elevated ACOX1 protein level characterized CAAT of women with malignant tumors, independently from obesity, while elevated ACADM protein level characterized only CAAT of obese women with malignant tumors. Furthermore, a higher rate of exosome secretion from adipocytes with a higher level of β-oxidation in cancer cells has been noted in obesity [28]. In light of these findings, light microscopy confirmed structural interaction between breast cancer cells and surrounding adipocytes in women with malignant tumors, especially pronounced in obesity. Fatty acids obtained by cancer cells can promote tumorigenesis via several mechanisms. Firstly, they can serve as building blocks during cancer cell proliferation or for the biosynthesis of lipid-signaling molecules that support tumor growth. Additionally, fatty acids may also present a notable energy source by translocating to mitochondria, where they are prone to β-oxidation. Energy surplus can also be stored in the form of lipid droplets as triglycerides or phospholipids, later used as energy reserves for tumor progression [6,31].

Further, upregulated de novo lipid synthesis is currently considered one of the hallmarks of aggressive cancer phenotype as cancer cells become more independent from external nutrient sources, consequently exhibiting increased tumorigenesis [31]. ACC is the first enzyme in lipid biosynthesis and therefore the critical regulatory point. The dual roles in tumor progression have been demonstrated for ACC [32]. On the contrary, the expression of the rate-limiting enzyme FAS is most likely to be increased in several cancer types. Specifically, studies on breast cancer [33,34,35] revealed that the inhibition of FAS leads to lower cancer cell proliferation and higher sensitivity to chemotherapy. Likewise, cancer cells yet rely on the uptake of fatty acids from TME. Correspondingly, our results demonstrated changes in the level of fatty acid synthesis. Increased FAS protein level was observed only in malignant tumor tissue of obese women, with the same increase trend in malignant tumor tissue of normal-weight women compared to their benign counterparts. In CAAT, both ACC and FAS had increased expression in women with malignant tumors compared to women with benign tumors, independently from obesity.

Altogether, there are clear differences in lipid metabolism reprogramming between normal-weight and obese women with benign or malignant breast cancer. Malignant tumor tissue of obese women showed a higher expression of enzymes responsible for fatty acid mobilization, β-oxidation, and de novo fatty acid synthesis. These obesity-related differences could be significant for the diagnosis and treatment since the expression of named enzymes [36,37,38,39,40,41,42] all correlate with the aggressiveness and progression rate of breast cancer [43].

The existence of preserved mitochondria in both benign and malignant tumor tissue and CAAT was demonstrated via electron microscopy, which aligns with our results on mitochondrial oxidative phosphorylation. Malignant tumor tissue was generally characterized by an increased protein level of ETC complexes and ATP synthase compared to benign tumor tissue. Thus, in line with Warburg, who stated that oxidative phosphorylation in cancer cells never contributes less than 50% to energy production [44], and numerous recent studies [12,37,45], the reprogramming of oxidative phosphorylation and glycolysis with lactate production in breast cancer cells occurs simultaneously in cancer cells as a part of a unique metabolic strategy. Concurrently, CAAT showed alterations in ETC relative to malignancy and obesity. CAAT of normal-weight women with malignant tumors showed increased protein level of ETC complexes (except Complex I) and ATP synthase compared to normal-weight women with benign tumors, while the protein level of Complex IV and ATP synthase was even higher in CAAT of obese women with malignant tumors. Additionally, the protein level of ETC complexes showed differences between normal-weight and obese women with benign tumors, with lower protein levels of Complex IV and ATP synthase in obese women. This is in accordance with previously demonstrated lower gene and protein levels of some ETC complexes [46], as well as lower respiration rates [47] in adipose tissue of obese patients. Thus, some ETC complexes showed obesity-dependent protein levels revealing the possible impact of obesity on mitochondrial metabolic reprogramming in tumorigenesis. Further, even though our results based on protein level measurement point to the malignancy-induced, simultaneous reprogramming of mitochondrial oxidative phosphorylation in tumor tissue and CAAT in breast cancer, more extensive functional mitochondrial analyses are needed to reinforce this statement considering different levels of regulation most likely involved in establishing this state.

The importance of mitochondrial dynamics in tumor sustainability also comes from examining mtDNA. Namely, Cavalli et al. [17] and Hayashi et al. [48] showed that breast cancer cells and HeLa cells depleted of mtDNA display diminished tumorigenesis, which is restored after mtDNA reintroduction. Additionally, the function and number of mitochondria are portrayed through the changes in MCN [49]. Several studies of breast cancer have shown specific mutations in mtDNA [50] and lower MCN [51,52,53,54], but the significance of these alterations is not completely understood. In contrast, other studies have associated breast cancer with higher MCN [55,56]. Thus, MCN most likely relates to the molecular subtype of breast cancer, as well as the level of progression, epithelial–mesenchymal transition, metastasis, and therapeutic resistance [57,58,59]. Our results showed lower MCN in tumor tissue of obese women with benign tumors compared to benign tumor tissue of normal-weight women. In addition, malignant tumor tissue displayed similarly low levels of MCN, independent of obesity. Changes in MCN were closely followed by the changes in the protein level of PGC-1α in tumor tissue. Conflicting results regarding PGC-1α were found in breast cancer, where lower gene expression of PGC-1α was affiliated with lower survival patient rate and worse disease prognosis [60], while in vitro PGC-1α activation stimulated mitochondrial biogenesis and OXPHOS leading to increased invasive and metastatic potential of breast cancer cells [61,62]. On the other hand, we found an increasing trend of MCN in the CAAT of normal-weight women with malignant tumors compared to the CAAT of normal-weight women with benign tumors. Next, we showed a significant decrease in MCN in CAAT of obese women compared to normal-weight women with malignant tumors, with the same trend in women with benign tumors. This is in correlation with data showing that abdominal adipose tissue in obese patients is characterized by MCN decrease [63]. In accordance with MCN and the expression of genes involved in oxidative metabolism, we also showed decreased PGC-1α protein levels in the CAAT of obese women compared to the CAAT of normal-weight women with malignant tumors. These results are in line with animal studies that showed lower PGC-1α expression and subsequent lower expression of genes involved in oxidative phosphorylation and β-oxidation in subcutaneous adipose tissue [64,65], as well as lower PGC-1α expression found in the adipose tissue of obese patients [66]. Thus, our results are consistent with numerous studies stating that obesity is in relation to mitochondrial dysfunction in adipose tissue.

## 5. Conclusions

Overall, breast cancer could be seen as a complex pseudo-organ that represents a specific niche in which the tumor exists in complex symbiosis with its microenvironment. In this study, we showed that tumorigenesis is based on parallel and synchronized reprogramming of both tumor tissue and associated mammary adipose tissue. In addition to well-established changes in glucose and lactate metabolism [8,9], the maintenance and reprogramming of mitochondrial oxidative metabolism are also central to the establishment of the malignant phenotype in breast cancer patients. Furthermore, substantial tissue-specific alterations in lipid metabolism appear to be the ground on which oxidative phosphorylation is maintained in the malignant tumor tissue. Although it is generally considered that mitochondria are impaired in malignancy, our results display the maintenance of structure and protein level upregulation of ETC complexes and fatty acid β-oxidation enzymes, suggesting the importance of mitochondria in breast cancer. Still, the precise role and the impact of mitochondrial metabolic reprogramming and obesity on breast cancer progression in premenopausal women is yet to be disclosed. Our currently ongoing analyses (transcriptomics, proteomics, metabolomics, mitochondrial respiration, ETC complexes activity) will further unravel the precise role of mitochondria in breast cancer metabolic reprogramming.

## Data Availability

The data presented in this study are available upon request from the corresponding author. The data are not publicly available due to ethical reasons.

## Supplementary Materials

The following supporting information can be downloaded at: https://www.mdpi.com/article/10.3390/cells13020155/s1, Figure S1: Western blot.

## Abbreviations

TME	tumor microenvironment
CAAT	cancer-associated adipose tissue
mtDNA	mitochondrial DNA
MCN	mitochondrial DNA copy number
PGC-1α	peroxisome proliferator-activated receptor gamma coactivator-1 alpha
BMI	body mass index
CS	citrate synthase
PDH	pyruvate dehydrogenase
PDK4	pyruvate dehydrogenase kinase 4
ACADM	acyl-CoA dehydrogenase medium chain
ACOX1	acyl-CoA oxidase 1
ACC	acetyl-CoA carboxylase
FAS	fatty acid synthase
ATGL	adipose triglyceride lipase
ETC	electron transport chain
