# Supplementary material for: Breast Cancer: Mitochondria-Centered Metabolic Alterations in Tumor and Associated Adipose Tissue
Source: Cells. 2024 Jan 15;13(2):155. doi: 10.3390/cells13020155 (PMC10814287; doi:10.3390/cells13020155)
Supplement: Supplementary file 1 [file cells-13-00155-s001.zip › cells-2694781-supplementary.pdf]

*Western blot*

**Supplementary Figure 1.** Images of whole blots against PDH, PDK4, CS, ACOX1, ACADM, ACC, FAS, ATGL, COX I, COX II, COX III, COX IV, ATP synthase, PGC-1$\alpha$, and $\beta$-actin for tumor and adipose tissue. Each blot is representative of three independent trials and shows three representative bands per group (normal-weight women with benign tumors, overweight/obese women with benign tumors, normal-weight women with malignant tumors, and overweight/obese women with malignant tumors). for tumor tissue and adipose tissue. Three bands per group shown here represent three samples obtained by pooling nine samples from each group before loading.

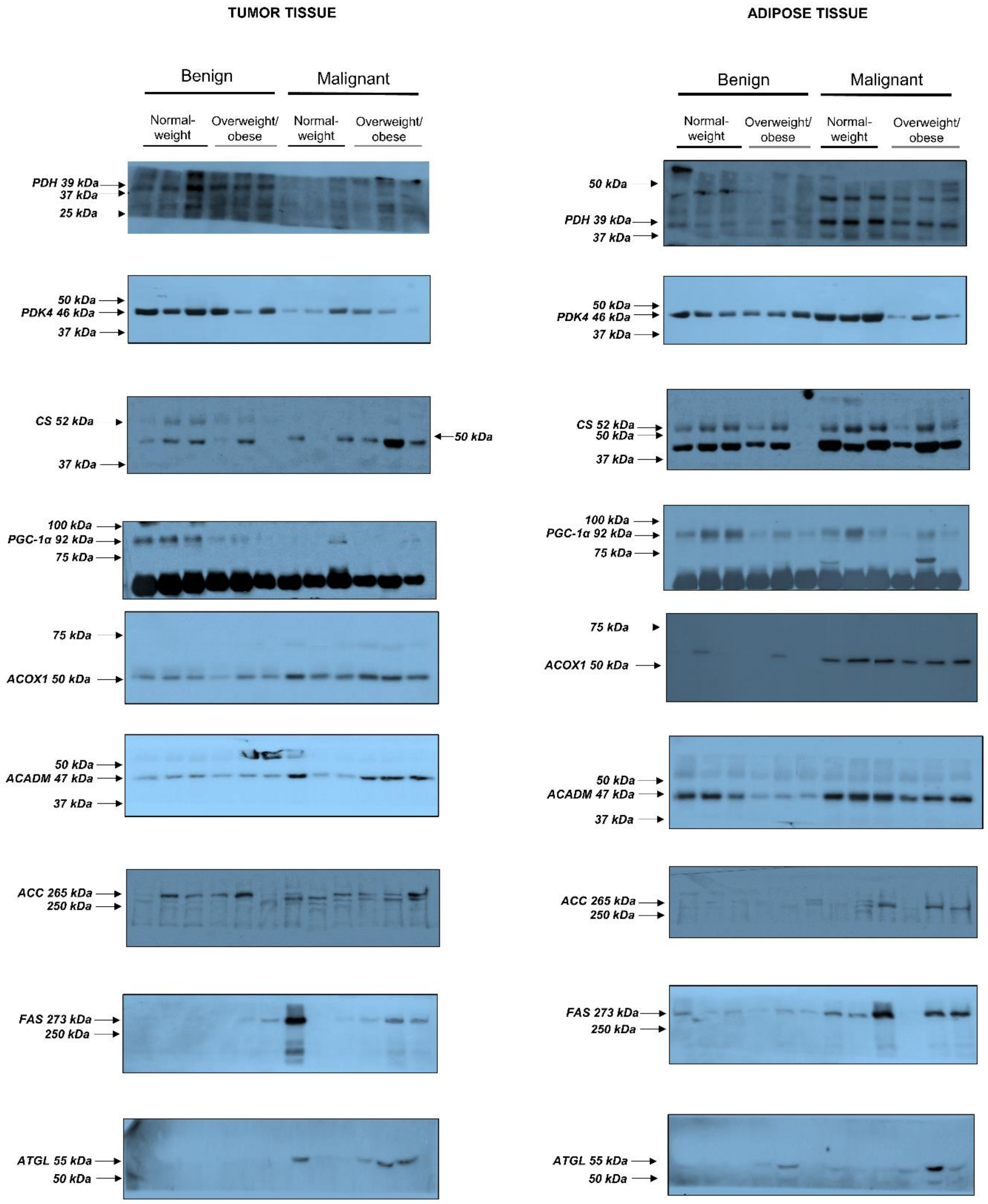

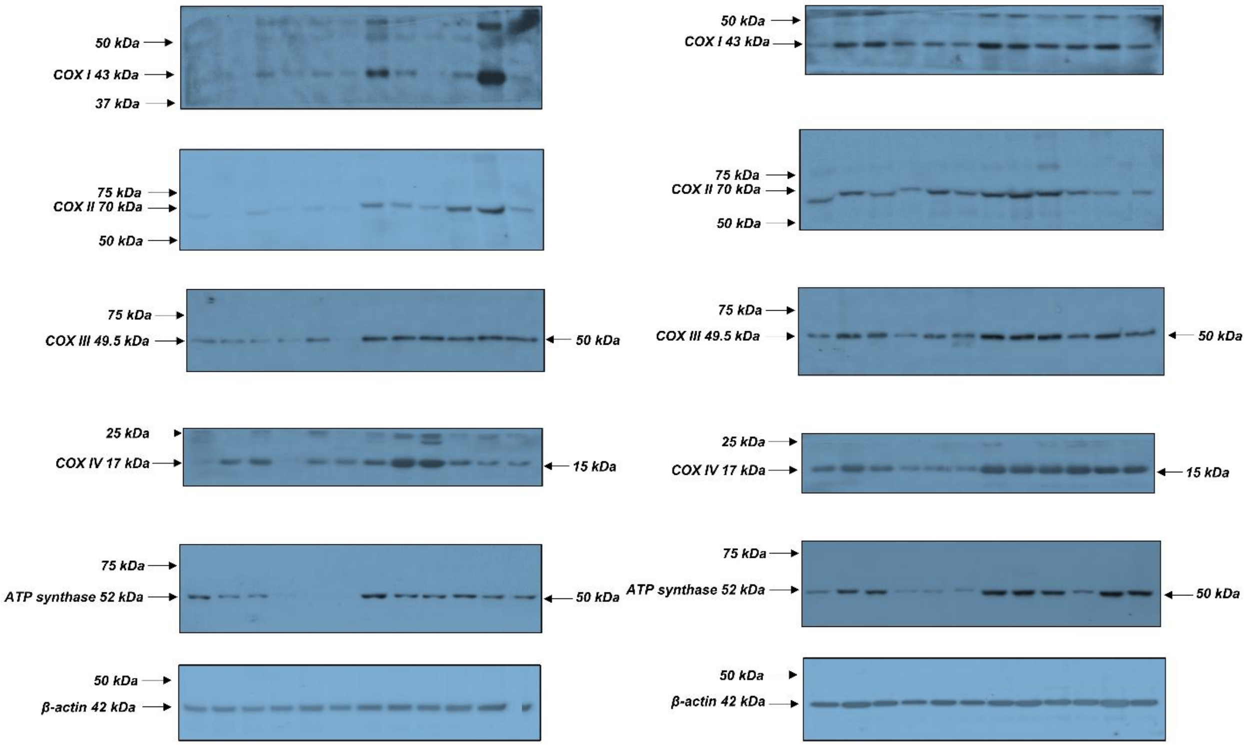
50 kDa
COX I 43 kDa
37 kDa
75 kDa
COX II 70 kDa
50 kDa
75 kDa
COX III 49.5 kDa
50 kDa
25 kDa
COX IV 17 kDa
15 kDa
75 kDa
ATP synthase 52 kDa
50 kDa
50 kDa
β-actin 42 kDa
50 kDa
COX I 43 kDa
75 kDa
COX II 70 kDa
50 kDa
75 kDa
COX III 49.5 kDa
50 kDa
25 kDa
COX IV 17 kDa
15 kDa
75 kDa
ATP synthase 52 kDa
50 kDa
50 kDa
β-actin 42 kDa
